# Men’s Help-Seeking for Distress: Navigating Varied Pathways and Practices

**DOI:** 10.3389/fsoc.2021.724843

**Published:** 2021-11-26

**Authors:** Alex Vickery

**Affiliations:** Centre for Research in Health and Social Care, School for Policy Studies, University of Bristol, Bristol, United Kingdom

**Keywords:** men’s help-seeking, distress, men’s mental health, masculinities, wellbeing

## Abstract

There is a widely accepted dominant narrative surrounding men’s mental health help-seeking, that men are less likely to pursue formal mental health support on account of hegemonic masculine ideals that limit emotional expression and vulnerability. Across the literature, little attention has been given to the varied ways in which men can and will seek out help when experiencing mental health troubles. This paper reports findings from a qualitative study of men’s experiences of distress, specifically focused on their help-seeking and everyday coping and management of distress. Between 2016 and 2017, 38 individual interviews were carried out in South Wales, United Kingdom, with men of a range of ages (21–74 years of age) and social backgrounds. Analysis identifies nuanced help-seeking practices and pathways, emphasizing ways in which men can and will engage with mental health support. Some men struggled with articulating personal issues in mental health terms, and some portrayed ambivalence to help-seeking, yet at the same time reconstructed help-seeking to positively align with masculine values. The paper further highlights the significant influence of familial and friendship networks in the help-seeking process as well as the value of therapy for men experiencing mental health difficulties, challenging the idea that masculinity inhibits the disclosure of emotional problems. Awareness of the diversity of ways in which men can actively engage with their mental health is needed so that mental health support interventions and practitioners can best reach out to men experiencing distress and provide gender-sensitive support suitable to a range of different men.

## Introduction

In recent years, the subject of men’s mental health has started to receive more academic attention. According to the Adult Psychiatric Morbidity Study (2014), women are still more likely to experience a common mental disorder (different types of depression and anxiety) than men, with one in five women self-reporting common mental disorder symptoms, compared to one in eight men (McManus et al., 2016). Yet, it is arguable that such data does not reveal the true extent of male mental health difficulties. Brownhill et al. (2005) argue that distress can manifest differently in men and as a result their expression of emotional difficulties can differ to women’s. Over the years, evidence has suggested that men are less likely to seek help or treatment from a professional for mental health difficulties on account of perceived threat to masculine norms (Courtenay, 2000; Moller-Leimkuhler, 2002; Addis and Mahalik, 2003; Galdas et al., 2005; O’Brien et al., 2005). As a result, large statistical datasets that explore prevalence of disorders and gender differences may not reflect the true level, or experience, of common mental health difficulties in men. For example, in 2019, men in the United Kingdom continue to be three times more likely to take their own lives than women (Office for National Statistics (ONS), 2020). This points to the complexity of men’s experiences of distress that belie the oversimplification of men’s behavior measured by prevalence rates. An understanding of men’s own lived experiences of mental distress, and the extent to which gender norms still impact men’s help-seeking behaviors have become an increasingly important topic of sociological interest. This is needed to be able move forward in engaging men in their mental health management and support those who might be particularly vulnerable to suicide.

Some authors maintain that much previous research on men’s mental health has occupied the “men as deficient” narrative and focusing on what men do not do in relation to their health and illness (Kiselica and Englar-Carlson, 2010; Seidler et al., 2016) rather than examining how men might be willing and able to engage in their own mental health and wellbeing. The research discussed in this paper aims to provide a nuanced understanding on how men engage in help-seeking for distress, rather than examine why men do or do not pursue support. In doing so, it adds to existing evidence about the relationship between masculinity and mental health help-seeking. The main aim of this paper is to examine how a diverse sample of men navigate help-seeking for broad mental health difficulties in everyday life and considers how masculinities can be practiced both negatively and positively to both restrict and facilitate mental health help-seeking.

## Masculinity and Distress

The social construction of gender can be understood as one of the most important sociocultural factors associated with, and influencing, men’s health related behavior (Courtenay, 2000). Through Connell (1995; 2005) social constructionist masculinities framework, gender is considered a configuration of practice through which social life is organized (p.71). Masculinity, or the now preferred term of masculinities, depicts the normative ways of being a man that can be constructed and enacted in various contexts of everyday social life (Connell, 1995). The notion of hegemonic masculinity has traditionally been referred to as the most culturally “honored way of being a man, that requires all other men to position themselves in relation to it: (Connell and Messerschmidt, 2005; 832). Yet, men in general may struggle to live up to the prescribed set hegemonic ideals (Courtenay, 2000; Moller-Leimkuhler, 2002; Chandler, 2012). Following this, multiple masculinities then emerge and compete. Thus, different kinds of masculinities that are vastly diverse, changing, differentiated and dynamic are present in all aspects of life. Robertson (2007) suggests that understanding masculinities as hierarchical “configurations of practice” that men move within and between provides a framework for exploring how and why men “do” health differently in differing social contexts (Robertson, 2007: 35). Over the past 2 decades, there has been an increase in qualitative research that explores men’s ill-health experiences, specifically how masculinity facilitates and inhibits behaviors and practices in health contexts (Robertson, 2007: 21). Courtenay (2000, 2003) influential work on masculinity and men’s health adapted Connell (1995) masculinities framework and demonstrated how by dismissing health care needs and health promoting behaviors such as asking for help and avoiding unnecessary risk, men are in fact constructing a particular form of dominant masculinity.

Constructs of masculinity can contribute to manifestation of distress in men. A study by Oliffe et al. (2010) found that the ways in which men embody depression in their everyday lives, for example through anger, isolation and risk-taking, can lead to symptoms of depression being interpreted as expressions of masculine ideals instead (Oliffe et al., 2010). Brownhill et al. (2005) “big build” model explained how masculine practices relating to depression, including avoidance, numbing, risk-taking and aggressive behaviors, could result in a path of destructive behaviors and further emotional distress. A study by Emslie et al. (2006) noted that during recovery from depression, it was important for participants to reconstruct a valued sense of themselves and their own masculinity (Emslie et al., 2006). Values associated with hegemonic masculinity (such as re-establishing control, being “one of the boys” and having a responsibility to others) were frequently incorporated into the men’s narratives of depression. Yet, they also found alternative patterns of expression, such as creativity, that challenged dominant forms of masculinity. Similarly, Valkonen and Hanninen (2012) found that masculine ways of thinking, and acting were also used as a way to facilitate coping with mental distress.

### Men, Masculinity and Help-Seeking

Health behaviors such as seeking out support have been seen to contribute to reinforce the active construction of gendered identities. By resisting support seeking it has been said that men are attempting to preserve manly self-reliance, control and autonomy and avoid appearing weak and vulnerable, practices of “hegemonic masculinity” (Connell, 2005). According to Addis and Mahalik (2003), men are less likely to seek help for a problem they see as unusual (e.g., mental health difficulties) and when they see it as a central part of their identity, for example, having emotional control is perceived as an element of masculine identity. Similarly, O’Brien et al. (2005) found that men who were most vulnerable were those with emotional or mental health problems, which they often interpreted as “stress” rather than admit to the “unmanly” diagnosis of depression (O’Brien et al., 2005: 515). Despite widespread reluctance to seek help amongst their participants, there were also instances where some men (e.g., firefighters) sought help as a way to preserve masculinity (because their jobs depended on sustaining good health and wellbeing) rather than dismantle it. Maintaining masculine practices can also be used in positive ways in relation to men’s help-seeking, and this should be considered when researching men’s help-seeking. Likewise, Farrimond (2012) argued hegemonic masculinity should be viewed with greater flexibility in relation to men’s health help-seeking practices, as her participants reformulated dominant masculinities in their accounts of help-seeking. Men in her sample negotiated masculine identities according to who was present (e.g., friends in similar situations, work colleagues), the type of illness and the identity demands of the social context. Farrimond (2012) argued that “taking action” (i.e., help-seeking) in relation to help-seeking was being reformed as a self-efficient, masculine act. However, Farrimond (2012) study focused on middle-class professional men who may have had better access to social tools that allowed them to reconstruct masculine values in this way. How help-seeking might be reformed or enacted for different men at different times warrants further attention.

The above studies examined help-seeking more generally, and there has since been much empirical research that has explore mental health, specifically seeking help for depression. Sierra et al. (2014) review reinforced the view that masculine attitudes and expectations of being strong and in control can increase men’s vulnerability to depression through perceptions of potential impacts to men’s masculinity, for example, in the case of help-seeking (Sierra Hernandez et al., 2014). However, like other studies (O’Brien et al., 2005; Emslie et al., 2006; Farrimond, 2012), when Sierra Hernandez et al. (2014) participants did seek treatment for depression, they redefined their masculine identity and perceived their help-seeking as retaining control, being responsible and actively dealing with their issues. Johnson et al. (2012) found that the gendered construction of help-seeking discourses took the shape of five discursive frames, with four of these discursive frames being variations on the dominant social discourse of men’s help-seeking and masculinity (i.e., men are often resistant to seeking help on the basis of masculine ideals) and one, genuine connection, reflects men’s willingness to talk openly about depression and how masculinity can be used in alternative ways to manage depression. Similarly, Siedler and colleagues’ (2016) systematic review found that conformity to traditional masculine gender norms impact on men’s attitudes, intentions and behaviors related to help-seeking yet they established that men would seek help if it were accessible and appropriately engaging for them. What would add to these important studies is a broader understanding on the help-seeking process and ways in which different men seek out both formal and informal support.

Such qualitative studies have been influential in discerning men’s gendered experiences of mental health troubles and how constructions of masculinity can influence help-seeking behaviors. Many have argued that much of the work examining men’s help-seeking experiences are somewhat limited because their sample populations mostly consist of single, heterosexual, white, employed, and well-educated men, who had already sought help for their depression and are willing to talk about their experiences to a researcher. Qualitative studies concerning help-seeking and men may miss out the experiences of socially diverse men, for example men of different socioeconomic status, sexuality and ethnicity, as well as the “silent” men who have experienced distress and emotional troubles and not sought out support. Furthermore, the tendency to focus on depression has tended to dominate the literature on men’s mental health and it would be beneficial to examine non-clinical samples of men who have experienced distress but may or may not have received a formal diagnosis.

### Defining Help-Seeking

Rickwood et al. (2005) describe help-seeking in response to mental health difficulties as “the behaviors of actively seeking help from other people. It is about communicating with other people to obtain help in terms of advice, information, treatment and general support in response to a problem or distressing experience” (Rickwood et al., 2005: 4). This paper makes use of Rickwood et al. ’s., definition of help-seeking through exploring the processes and various pathways in which men may go about seeking out support for emotional difficulties and troubles in living. Diverse sources of help include informal help-seeking from personal social relationships and networks such as family and friends, and formal help-seeking from professional sources of help such as mental health and health professionals.

Diversity in the behaviors of different men across various mental health difficulties, context and time require recognition. Help-seeking should be viewed as an interactive, ongoing process of formal and informal support seeking (Wenger, 2011: 495). In this respect, this paper considers help-seeking in a more nuanced way that acknowledges the process of help-seeking as being influenced by perceptions, interactions, skills and strategies, and changeable approaches and outcomes.

Findings presented in this paper are from a doctoral study of men’s experiences of help-seeking and everyday coping and management of mental distress (2015–2019). The overarching aim of this paper is to examine how the help-seeking process might look for different men, with the research question asking: what are men’s experiences of engaging in help-seeking for distress?

## Materials and Methods

Using a cross-sectional, qualitative design 38 men were interviewed, recruited from both the general population and mental health support groups. The study was given ethical approval from The School of Social of Sciences’ research ethics committee at Cardiff University.

### Recruitment

Between 2016 and 2017, 38 men in South Wales, United Kingdom (UK) were recruited through purposive sampling, seeking out men from both the general public and specifically men who attended support groups for mental distress. The reason for two sample groups was that the study aimed to examine both formal help-seeking (such as use of support groups and mental health services) as well as the more informal routes of help-seeking and everyday coping mechanisms. In addition, the purpose of recruiting men from the general public was to seek diversity within the sample by age (range 21–74 years) and socio-economic status. Men who self-identified as having experienced distress were recruited because the research was interested in including men with and without a formal diagnosis of a mental health problem. This was so that the research could elicit a broader understanding of help-seeking in men’s everyday lives. The paper will discuss mental health and distress as broadly defined. Defining “mental health” can be controversial as well as problematic because there are markedly different ways of speaking about mental normality and abnormality in today’s society (Rogers and Pilgrim, 2010). The author acknowledges that there are many different terms used today, for example, “mental health,” “mental ill-health,” “mental well-being,” “mental health problems,” “mental distress” and “emotional difficulties.” The author uses the term distress and emotional difficulties, defining it as a challenging emotional experience (e.g., anxiety, low moods, stress, isolation). According to Holland and Blutz (2007) distress can happen in a range of severities, which may not lead to a clinical diagnosis of mental health problems. Men with severe mental illness (such as schizophrenia) were excluded from the study as the focus was on common mental health difficulties such as depression, anxiety, and non-clinical emotional distress. Also, those under a current psychiatric care plan or at any perceived risk were not included in the study.

Two different recruitment fliers (for men using support groups and men from the general public) were created and distributed, briefly describing the study and inviting men to contact the researcher to take part. To access men who had attended support groups, facilitators of third sector and voluntarily attended support groups acted as gatekeepers and were contacted first with the recruitment flier, asking if the researcher could initially attend a group to talk about the study. Following this, men from support groups who were interested in taking part were able to speak to the researcher and subsequently arrange an interview. To recruit men from the general population, the researcher distributed fliers in various public locations and institutions, specifically institutions perceived to be those where men frequented, such as pubs, bars, betting shops, barbers, sports clubs and some workplaces. The researcher approached men with the researcher flier inviting them to take part in the study and in most instances the researcher obtained contact details and interview arrangements at that initial time of speaking. Snowballing also took place through participants already recruited.

Table 1 shows the sample of participants, with their ages and classified socio-economic status, using the three-class version of the National Statistics Socio-economic classification (NS-SEC measurement). Despite the recruitment aims and efforts, most participants were White British, with two participants being non-White. This is a limitation of the study, mostly likely due to the population demographics of South Wales, where the recruitment took place, despite attempting to access inner-City locations that might be more diverse in terms of ethnicity. Two men in the general population sample identified as gay. Information about participants’ mental health status was not officially collected using a written questionnaire but the focus of the interview schedule included questions asking participants to recall their experiences of mental health difficulties. The researcher endeavored to ensure participants felt comfortable retelling their experiences of distress during the interview, keeping it relaxed and conversational to create a comfortable space for men to share their experiences. Similarly, general health status was not formally collected but questions around general health and any experiences of broader health issues were asked during the interview. Pseudonyms were assigned at the interview stage to protect participants’ anonymity.

**TABLE 1:** Sample groups and participants.

General public participants *n = 19* age range: 25–74	Support group participants *n = 19* age range: 21–74	Occupational category (NSSEC three class version measurement)
John (41), Simon (42), Oliver (36), George (45), Jake (29), Daniel (34), Harry (65)	Thomas (62)	1. Higher managerial, administrative and professional occupations
Dave (27), Nick (56), Shaun (48), Colin (retired, 74), Nathan (49), Kevin (retired, 65)	Kyle (34), Richard (24), Peter (retired, 62), Joseph (retired, 68), Andrew (retired, 68), Mark (retired, 64), Jim (retired, 74), James (retired, 61), Rhys (57), Samuel (29), Patrick (54)	2. Intermediate occupations
Geoff (54), Jason (55)	Albert (51), Ben (52)	3. Routine and manual occupations
Adrian (59), Steven (52), Joel (33)	Barry (38), William (44), Matthew (54), Adam (48)	^a^Never worked and long term unemployed
Mike (25)	Rick (21)	^a^Student

a Residual operational categories: When using the three-class version “Never worked and long-term unemployed” and “Student” are not classified within a category.

The author, and sole researcher, carried out individual in-depth, semi-structured interviews at a time and public location convenient to the participant, which included a university room, various coffee shops and local pubs. While there were some ethical concerns with conducting interviews involving sensitive content in public places, researcher safety was of a particular concern as interviewing men in their own home can be potentially dangerous for a lone female researcher. To overcome this, the researcher offered participants the choice of public space that they felt comfortable with for the interview to be held. Written informed consent was gained prior to the interview with ongoing consent sought verbally throughout the interview. An interview topic guide was used, which differed slightly for the two different sample groups of men and included “ice breaker” questions about themselves and their lives, questions on any experiences of distress and emotional difficulties, questions on help-seeking, questions about everyday informal coping mechanisms when experiencing distress, and specifically for the men who had accessed voluntary groups, questions about their use of support groups for distress. At the end of the interview all participants received a resource sheet of mental health services, and the researcher had a safeguarding protocol in place for terminating the interview should any participants have become distressed during the interview. On occasion, when some participants became visibly upset, the researcher paused the interview and asked if they wanted to take a break or finish the interview.

### Data Analysis

The interviews were digitally recorded and transcribed verbatim by the author, which assisted the researcher to become fully emersed in the data. Thematic analysis was used since it lends theoretical freedom and flexibility (Braun and Clarke, 2006). The researcher coded the transcripts using NVivo version 10.2, taking both an inductive and deductive approach (Strauss and Corbin, 1990). This involved having a coding list of predefined, deductive codes based on constructionist masculinities theory (e.g., traditional beliefs, masculine language) and literature on men’s mental health and help-seeking (e.g., barriers to help-seeking), supplemented with codes that arose inductively. Following repeated reading of the interview transcripts, initial themes and codes were derived and the researcher began coding broadly on the general population sample dataset first. Some data segments had several different codes assigned to them and codes were then combined to create themes. Themes can be identified by, “bringing together components or fragments of ideas or experiences, which are often meaningless when viewed alone” (Leininger, 1985: 60). Data was compared within and across the interviews from both sample groups and continued to allocate descriptive codes to data segments. Initial themes and thematic maps were then reviewed and refined to include the merging of the two sample groups.

Theme definitions were produced and validated by returning to check on the coded data and re-read extracts. The aim was to interpret the meanings and significance underpinning each theme and move from a descriptive to an interpretative level. To do this, the researcher used Connell (1995) social constructionist framework of masculinities to interpret participants’ mental health seeking practices in relation to masculinity as well as applying new literature relating to emerging themes. Ways in which participants drew upon, and moved within and between, different patterns of masculinities when articulating their experiences were interpreted using the constructionist gender framework and incorporated into the examination of men’s nuanced help-seeking practices more broadly. Data analysis was carried out solely by the author with support of two research supervisors who reviewed some coding frames, thematic maps and theme descriptions with data extracts and chapter drafts to ensure rigor and reliability of the analysis.

## Results

A total of 38 men aged 21–74 years participated in the study. In contradiction to the idea that men do not seek help, most participants from the general population sample had also sought, or at least attempted to seek out, some type of formal support for mental health and emotional difficulties. Analysis revealed that participants’ help-seeking practices were not straightforward, and their navigation of help-seeking routes and types of treatments were complex and varied, depending on individual circumstances. Nevertheless, there were six broad themes identified across the accounts regarding practices of help-seeking: 1. Using other terms to articulate distress; 2. Ambivalence around help-seeking; 3. The importance of significant others; 4. Influential cultural milieu; 5. Reframing help-seeking, and 6. The benefits of talking to others. These themes focus on a more nuanced understanding of ways in which men speak about mental health help-seeking.

There were broad themes identified across the accounts from all participants around their varying practices of help-seeking. There were not clear distinctions in help-seeking practices between the two different sample groups and support group attendance often came after speaking to the GP or attempting to seek out talking therapies. For this paper the focus will be on initial help-seeking behavior and the nuanced ways in which men speak about help-seeking practices and pathways are examined within these themes.

### Using Other Terms to Articulate Distress

Some participants spoke of interpreting, and articulating, symptoms of distress (such as fatigue, low mood, restlessness and irritability) as something other than mental health difficulties, which impeded seeking out professional help. Oliver spoke of his experience when he was at University in his early 20’s and appeared to interpret his emotional difficulties as a physical issue:

Interviewer: Did you go to the GP off your own back?
Oliver: Yeah, it was just, it was sort of an on-going [thing] because I was becoming, I wasn’t really sleeping well. I was very, very restless and anxious all the time and I perceived it as some kind of physical problem and my GP was like no I don’t think so (Oliver, 30–34 years. General public sample).

As a result of this difficulty interpreting distress symptoms, participants were reluctant to articulate personal issues as mental health difficulties, which could be a way of protecting their masculine selves. Oliver’s experience aligns with traditional beliefs that for men, physical illness represents a legitimate reason for accessing health services whereas struggling with aspects of daily living might not warrant professional help due to the stigma still attached to it. Articulating distress using different terms to a General Practitioner (GP) revealed symptoms that generated discussion of the less preferred topic of “depression.” Similarly, Nick had previously experienced cancer and as a result was out of work for some time (although was working at the time of interview). The researcher asked about how that time affected him emotionally and he described a previous time consulting the GP for “something different” but leaving the appointment with anti-depressants, having been told that he was experiencing depression:

Interviewer: Did you go to the doctors about depression when you were feeling low or during those times?
Nick: No, no. I was probably more depressed when I split up with my son’s mother, which was years before then. I did see a doctor then, not about it, he just knew.
Interviewer: Were you going for some other reason?
Nick: Yeah, I can’t remember. He was a locum; he wasn’t a regular. He was just there for like a fortnight and he said, “I think you’re depressed” and he said, “are you?” And I told him, and he gave me some tablets (Nick, 56–60 years. General public sample).

When Nick was asked how he had replied to the GP after being asked if he thought he was depressed, he said “Yeah, I did say yeah. I can’t remember why I went there. I think I was like low and like tired all the time and everything like that and he said he sort of just knew. He’s the only one that’s ever said it to me” (Nick, 56–60 years).

Nick’s reluctance to articulate his personal issues as mental health difficulties points to how dominant discourses around men’s mental health are still very much present. Hegemonic practices that limit emotional expression shaped the initial help-seeking process for these participants as they attempted to avoid vulnerability by using alternative ways to articulate experiences of distress. In minimizing the severity of their distress experience and the magnitude of need for help, the men could protect masculine identity and any potential for loss of self-esteem in being given a psychiatric classification (Johnson et al., 2012: 352). Nevertheless, by initially presenting to the GP (albeit not explicitly for emotional distress) these participants recognized that something was wrong and acknowledged the need for some assistance. This points to the counteracting of hegemonic standards that have traditionally inhibited men from seeking professional help, pointing to the ways in which different men, dependent on context, can engage in various behaviors typically associated with different masculine ideologies.

In connection to using other terms to articulate distress, two participants explicitly noted how in discussion of mental health and support seeking, men are inclined to associate any kind of emotional difficulties under the expression of “stress” instead:

Ah I don’t know anybody that talks about it in those terms really. It’s just like “ah you know I’m happy, a bit stressed.” Stressed is the classic catch all isn’t it, lump everything under stress, yeah, a bit low or they’ll talk about a particular issue, “like you know me and the missus are not getting on” or you know, “I don’t know what the future holds you know.” […] If you’re having an emotional, relationship issue, you perhaps pile all those issues into one thing. Nah blokes, my friends at least, don’t really talk in those terms (Simon, 40–45 years. General public sample)

Mike said similar about men articulating and framing mental health difficulties as “stress”:

I think one of the words that’s kind of been normalized, which it shouldn’t have been, particularly in men, is stressed. “Ah I’m really stressed about it, ah I’m stressed.” Well, that used to mean, stressed used to mean unhappy and nowadays if you said, “I’m actually really unhappy at the moment you know, work’s bad, I’m really unhappy,” that would be weird. To say, “I’m stressed,” it’s kind of the norm, it’s like “oh yeah, everyone’s stressed aren’t they” and that gets used a lot. I think a lot of men in particular use that as a kind of guard, they say “aw I’m pretty stressed at the moment.” They really mean, “I’m anxious and I’m tired, I’m angry and sad.” That’s a word they use to deflect from mental health, I think (Mike, 20–25 years. General public sample).

These accounts suggest that amongst groups of men, the admission of “unmanly” depressive symptoms might still carry with it fears of shame, vulnerability, and societal stigma (O’Brien et al., 2005; Emslie et al., 2006). The quotes above reveal how using the term “stress” might be acceptable within men’s lives and conversations with others and may instead denote feelings of manly self-reliance and success through the notion that men become stressed through paid employment and the pressures of providing.

### Ambivalence Towards Professional Support

In the experience of seeking out mental health support, several participants noted that cost, lack of time and availability of services, in particular waiting lists for counselling services, were practical hurdles participants spoke of having to initially navigate. Daniel explained that he sought counselling from a professional after a friend advocated the benefits of talking to a counsellor, however, Daniel’s response was “but it’s finding time.” He appeared ambivalent in his efforts to seek out formal support:

It’s quite ironic that I’m doing this because about 3 months ago I just woke up and I had, I wouldn’t call it a panic attack, but a bit like an anxiety attack. So, I tried to go to a psychiatrist, a psychologist. She wasn’t able to fit me in, so I haven’t gone. Yeah, I rung this woman up and typical man, sort of you know, can you fit me in, and she was like “no I can’t take anybody on.” Oh, forget it (Daniel, 30–35 years. General public sample).

In portraying his ambivalence, Daniel related help-seeking to wider discourses of masculinity through generalisations of what “typical men” do, seemingly to defend his reasons for not further seeking out professional help. What is noticeable here is that Daniel did make effort to ring the psychologist (having been encouraged by his female partner) yet was faced with the obstacle of a waiting list. This conundrum challenges the dominant discourse that men are reluctant to seek out help and instead recognises obstacles beyond men’s control that can potentially impede men seeking support.

Another participant also discussed his intention to seek formal help for emotional distress but again implying an ambivalence, said he changed his mind due to the prospect of waiting lists:

Andrew: But I did think once of asking the doctor if I could see a psychiatrist. Which would have to be a waiting list for it. The only service I had to have, was to get myself going, to up-service myself. I couldn’t ask anybody else to sort my problems out for me. I had to do it myself.
Interviewer: But you were thinking about asking?
Andrew: I was thinking about it at the time, yeah. Psychiatrist was on the book see but alas a time went by and that’s it (Andrew, 66–70 years. Support group sample).

For Andrew, it seemed to be a combination of both availability of an appointment with a professional, the requirement for self-control of his own health (Robertson, 2007), as well as confidence in his own masculine self-efficiency that contributed to this. By noting how it was his job to sort himself out, without the help of anyone else, he positions himself in line with traditional ideals of masculinity, such as self-reliance, stoicism, and independence. This degree of ambivalence presented by these participants could be due to the type of help that they perceived as available to them, and the idea of waiting lists and relying on someone else for support which may threaten masculinity.

### Attempting to Reframe Help-Seeking as “Normal”

Some participants’ attitudes and assumptions surrounding mental health help-seeking altered throughout their accounts when retelling their experiences. James realized this change within himself and his perception on seeking mental health support. James had previously sought help from the GP which led to one-to-one counselling, something that he identified as particularly effective:

It sort of just changed something for me. Sort of said, yeah if I break my leg, I go out to a doctor. It’s the same thing. You need help, you need professional help, go see a doctor … (James, 60–65 years. Support group sample)

Participants who generally had more positive experiences and perspectives towards seeking professional help reframed the help-seeking process, attempting to normalize it. James’ comparison of emotional distress to breaking a leg, in the way that it should be treated, highlights the way men might privilege the physical over the emotional. In response to potential vulnerability, he attempts to reconstruct traditional masculine ideals to view help-seeking as an active, positive, and strength-based perception action. Such comparisons were seen within other men’s narratives in attempt to reframe and normalize mental health help-seeking through physical metaphors, and positioning disclosure of distress to actively deal with the issue:

You know, I have a golf lesson once a month just to tidy up my golf swing, it’s exactly the same as probably going to see somebody. Just talking through your issues, which is life (Daniel, 30–35 years. General public sample).
If my pipes are leaking in the house, I don’t just shut the bathroom door and hope it’s going to go away. You know, you phone a plumber and get an expert in. But you know we don’t do that do we, well men don’t do they? (Simon, 40–45 years. General public sample).

These men showed awareness of the importance of seeking mental health support, pointing to a general, and more positive shift in hegemonic values to do with mental health and help-seeking, allowing for men to positively engage in help-seeking. Nevertheless, reframing help-seeking in this way and relating it to physical, masculine practices that concern “fixing things” may actually be reinforcing hegemonic masculine values. Simon also acknowledged the dominant discourse that men do not seek expert help. Simon started by saying “but you know we don’t do that do we” but then restructures his sentence by saying “well men don’t do they.” Using the homogeneous terms “men” and “they” in his narrative, Simon attempted to distance him away from masculine stereotypes, as he had sought out counselling himself. Despite associating help-seeking with a sense of masculinity and reframing it in masculine terms, in articulating their experiences to the researcher, these participants purposively attempted to position themselves outside of the dominant narrative that men cannot and will not seek mental health help.

### The Importance of Significant Others

Most participants who had sought formal help for distress explicitly acknowledged the importance of a significant other in the decision to navigate and seek out formal help. Through initially talking to someone close to them (including partners, parents or close friends) about emotional difficulties, participants were encouraged and supported to seek out more formal support, usually from the GP. Two middle aged men had spoken to their mothers about their emotional troubles who aided their help-seeking, Nathan said “I spoke to mum about it and that and I realized you know it was the best thing to do, to explain” (Nathan, 46–50 years, general public sample) and Steven said “I spoke to my mother I think, and I think it was mum who said you know just go and speak to somebody” (Steven, 56–60 years, general public sample). For participants who had spoken with professional services, initially disclosing distress to family and friends put them in a less vulnerable position and was an influential starting point that promoted formal help-seeking, as Samuel explained:

It started off with friends, then parents, then the GP and then who they referred me to. Whereas I don’t think I could have gone straight to the GP or the services. I think I needed to make that admission to somebody else I was comfortable with first and then that would have given me time to sort of process that and say yeah, I do need help (Samuel, 26–30 years. Support group sample).

There are different pathways to doctors, and people often use “lay networks” of friends, families or other individuals to help them assess and respond to symptoms (Smith et al., 2005).

### Influential Cultural Milieu

Participants’ help-seeking behavior was also influenced by what they perceived to be the norm within their own cultural milieu. In some men’s accounts, having friends who had sought help for distress further motivated formal help-seeking:

Interviewer: Did you go to the doctors off your own back?
John: Yeah. It was funny because I was talking to another friend. He was having something similar, and he described it to me as he just didn’t know what to do for help and this depression was like a big wall in front of him and he didn’t know how to get over the wall. He said he went to the GP and started just the talking therapies, and he took medication, and he described it as the wall started to come down and he started to see a way over it, and how to move on and that’s exactly how it felt to me. I went to the GP, and I was really surprised, I just burst into tears and just sort of off-loaded everything which came as a–I didn’t expect to do that. I expected to go have a much calmer conversation with him (John, 40–45 years. General public sample).

Seeing other men resisting hegemonic masculinity and disclosing details about their mental health and help-seeking can be so profound that it gives it legitimacy and permitted other men to also resist hegemonic masculine ideals. Specifically, John’s account indicates the influence of descriptive masculine norms that are produced when a male observer sees what other men are doing in a situation (Addis and Mahalik, 2003: 10). When disclosing vulnerable emotions and feelings, men may hear important (male) others say that it is important to get help for distress or also observe men they know receive help. Seeking help and divulging personal experiences became normalized within John’s social group (John is a gay man with a circle of male friends) and influenced his help-seeking behavior through social norms reproduced within that group.

### Talking to Others on Their Own Terms

Many participants (10 of 19 men interviewed from the general population and 15 of 19 of men from the support group sample) had engaged in some form of talking therapy in the past. Some participants had accessed counselling through their workplace or university, others had been referred to services through their GPs and a few had sought out private counselling themselves. These men constructed the desire to just have someone listen. Some men recounted a preference to speak at length with a therapist over speaking to a relative or friend, which sits in contradiction to the theme that describes the importance of significant others in men’s help-seeking pathways. For Shaun, it was having someone independent of the situation acknowledging the extent of his distress:

To have somebody else say “yes, that’s a shed load of shit” was cathartic enough for me to be able to contextualize, probably stuff that went way back to when I was a teenager. So that really helped me, just for somebody else to say, “yeah, that was difficult, it’s no wonder you felt like that” (Shaun, 46–50 years. General public sample).

This relationship, whereby they are the ones choosing to disclose personal emotions, also allows participants to construct themselves as active and empowered agents in their relationships with formal support providers (Johnson et al., 2012). These participants did not completely abandon masculine ideals in their preference for talking, as making the decision to seek out therapy and knowing what they want out of that relationship signifies independence and autonomy. This also demonstrates men’s resistance of hegemonic ideas around men and help-seeking and highlights a pursuit of an alternative masculinity that embraces and recognizes the value of disclosing emotions, albeit on their own terms. Additionally, it was preferable to speak to a third party, as someone who “won’t offer an opinion” (Daniel, 30–35 years, general public sample). Oliver, also felt similar about speaking to a therapist:

I think you’re not feeling like you’re burdening someone. If you talk to a friend, it can feel very much like you’re giving someone else your problems to deal with whereas when you’re talking to a professional, I felt much less guilty (Oliver, 30–35 years. General public sample).

Feeling like a burden or wanting to talk to someone independent is not necessarily distinct to men’s gendered experiences of mental health support. These men, however, specifically perceived therapists as having detachment to their situation and observed them as having a job to do, precipitating more comfortable disclosure, and lessening any resulting shame or guilt. This theme notably challenges the dominant discourse surrounding men and the disclosure of emotions.

## Discussion

In recent years, men’s mental health experiences have gained much more research focus, exploring the complex relationship between masculinity and men’s health-related behaviors, including help-seeking. The findings in this paper build on research exploring men’s mental health experiences, help-seeking, and its relation to masculine ideals, and further identifies more nuanced understandings of help-seeking practices and pathways that men might engage in. In an effort to move away from focusing on masculinity as a determinant of whether men do, or do not, choose to seek help, the paper prioritizes instead the different ways men can engage with mental health support seeking. The study provides a new contribution to the under-researched field of men’s mental health by focusing on help-seeking of men who may, or may not have, received a formal diagnosis, thus moving beyond an often-sole focus on depression, and using a distinct sampling procedure in attempt to include more diverse experiences.

There are several important findings. First, men’s participation in this study, and the emerging themes, support other studies that have found that some men do seek help for distress under the right circumstances and if it is accessible and engaging (Cheshire, et al., 2016; Seidler et al., 2016). Nearly all participants had sought help or at least attempted to seek help for some troubles in living, even if they had not pursued it further or utilized continued mental health support. The paper highlights the need to acknowledge the varied and positive ways in which men can, and do, manage their own mental health, as well as noting the diversity of informal and formal help-seeking practices engaged in. This supports the argument made by Keohane and Richardson (2018) that instead of examining men as resistant to mental health help-seeking, we should question their ambivalence depending on the type of help available and the context in which they seek formal help (Keohane and Richardson, 2018: 167).

Second, the findings presented emphasize connections between masculinities and masculine expectations, and men’s different help-seeking behaviors and practices. The first theme “using other terms to articulate distress,” reveals that men might still have difficulty in recognizing and interpreting mental health symptoms and they may initially show reluctance to name and disclose personal issues as mental health difficulties, not identifying their experience as a pattern of mental health help-seeking. Articulating personal struggles as mental health difficulties might pose risk to hegemonic masculinity (O’Brien et al., 2005) and in communicating distress in a different way, not directly seeking out mental health help, these men attempted to guard their vulnerability, retain a sense of control, and protect their masculinity and masculine status (Johnson et al., 2012). Participants described how men might be more likely to use the term “stress” to articulate more personal troubles and this could be interpreted as an attempt to hold on to masculine status in times of struggle. However, the notion of “stress” could also be considered a statement of vulnerability but being perceived as a temporary and situational based vulnerability, it carries less stigma of ongoing weakness than traditional diagnostic terms. The men in this study who used different terms to articulate emotional troubles did however seek out some professional help, and this should be acknowledged despite them not explicitly recognizing it as a help-seeking pattern for psychological terms. This also points to the important role GPs play in recognizing and diagnosing distress, such as depression and anxiety, in men when they might articulate it in different ways (Cheshire et al., 2016).

Relating to the above, the theme “ambivalence around help-seeking” further points to how men may attempt to preserve notions of masculinity by deciding not to seek help, despite recognizing the need for it. This aligns with Robertson (2007) “should care/don’t care” dichotomy that argues that because society values healthy practices and health as a moral responsibility, men must balance the masculinity requirement of showing that they do not care about health with the opposite belief that they should. However, as noted in the results, these men depicted a sense of confidence in their self-management of distress, emphasizing masculine identity through self-efficiency yet also portraying that they were willing to take responsible action for their mental wellbeing. Observed in the first three themes, participants drew upon discourses and values associated with hegemonic masculinity (Jeffries and Grogan, 2012; Johnson et al., 2012) when articulating their experiences. For example, one man describes having to sort his problems out himself and “service himself” after contemplating seeking professional mental health support, aligning with notions of independence and autonomy. This same participant was attending a community support group aimed at supporting the wellbeing of older men, thus highlighting a different pattern of help-seeking. The theme “reframing help-seeking” shows how participants reconstructed help-seeking practices to align with hegemonic masculine values and behaviors such as self-efficiency and taking responsible action to manage issues (Farrimond, 2012; Johnson et al., 2012; Sierra Hernandez et al., 2014). In normalizing and reframing help-seeking, these participants constructed themselves as active participants in seeking mental health help (Johnson et al., 2012) and drew on notions of hegemonic masculinity in more positive and proactive ways.

Third, the findings demonstrate how help-seeking is a process of engaging with different support opportunities in a variety of contexts, as highlighted in the themes “the importance of significant others,” “influential cultural milieu” and “talking to others on their own terms.” Wenger (2011) argues that we should view help-seeking as an interpretative process in which a recognized need is identified and in doing so, we should consider how men work with others in their lives to make sense of their needs. For men in this study, encouraging committed partners were prominent in men’s help-seeking practices, whilst allowing them to maintain their male identity through legitimizing help-seeking. The influence of others in a man’s social network (Addis and Mahalik, 2003), in particular seeing other men resist hegemonic masculinity and seek out mental health help, was so profound that it legitimized help-seeking and allowed these men to break down some masculine stereotypes and stigma attached to help-seeking. The social norms model (Sieverding et al., 2010) suggests that seeking help is influenced by what is commonly approved by important others and what is commonly observed as done. It is important to consider here though that where men’s social groups hold more traditional and/or negative masculine norms about help-seeking and mental distress (e.g., “men do not get depression”), the influence may be just as profound and lead to resistance to seeking out support. The example of John presented in the data (a middle-aged, well-educated, gay man), who sought help following a conversation with a male friend, also points to the importance of noting the intersection of other social characteristics (socio-economic status, sexuality) and their influence on social group norms and men’s behaviors.

The final important finding is that participants also renegotiated the performance of hegemonic ideals around men and help-seeking and placed value on the importance of talking about distress. The theme “talking to others on their own terms” points to men’s active pursuit of an alternative masculinity that embraces connection and disclosure, albeit on their own terms. This supports previous literature that suggests the ways in which men can resist hegemonic masculine ideals and embrace alternative patterns of masculinity which enable them to positively engage with their mental health (Farimond, 2012; Johnson et al., 2012; Sierra Hernandez et al., 2014; Seidler et al., 2016). Prioritizing therapy and disclosing distress on their own terms allowed for men to incorporate a sense of hegemonic masculine values in an alternative way that allowed for open and sensitive disclosure to manage distress. This also highlights a shift in hegemonic standards over time, whereby it might now be more acceptable for men to openly discuss distress and help-seeking, content, and context dependent (Chandler, 2021).

### Implications for Practice and Limitations

By not applying a narrow, deficiency approach of what mental health help-seeking entails, the findings gather more nuanced insights into the help-seeking process and how it might look for different men in their everyday lives. Furthermore, by recruiting men from support groups, the research has also considered the reasons and motivations for why men do seek help for distress, and specifically the kinds of support that men might utilize. The attention to the diversity of ways men do seek help may assist healthcare professionals and practitioners to develop strategies for engaging men. Understanding how others can influence men’s help-seeking behaviors can support guidance for how to facilitate help-seeking relationships among male clients and also inform tailored interventions for promoting men’s mental health. The findings could inform such interventions by emphasizing the importance of positive social support relationships for assisting men to seek help and through drawing attention to the ways in which men can alternatively use masculinity to positively manage their mental health in their everyday lives.

Despite the above, the research comes with limitations. This paper has focused on professional mental health help-seeking, such as speaking to the GP or counselling, as well as informal help-seeking through family and friends, and it is important to acknowledge that men may also use other sites of help such as web resources or helplines, when experiencing distress. Furthermore, the sample consisted of atypical groups of men from South Wales, who were willing, and able, to talk about their distress experiences at length with a researcher. Men with experience of distress and more positive attitudes to help-seeking are more likely to volunteer to take part in research than those with negative views and experiences of mental health help-seeking. The study attempted to access the more “strong and silent” men through the unique recruitment procedure and the sample did consist of men who described how they had never opened-up about their experiences until the research interview. It was found that the female gender of the researcher and the confidentiality aspect of the research interview setting positively influenced men’s willingness to share more personal experiences. On the other hand, this might have also had influence on the ways in which participants positioned themselves in relation to masculine discourses in the presence of a female researcher, as they knew the research was interested in experiences of distress *as a man,* which could lead to self-conscious performances of masculinity*.* For example, men may have tried to assert some masculine dominance in directing the conversation in attempt to minimize vulnerability. In addition, the sample lacks ethnic diversity, even with the attempt at recruiting men of diverse social characteristics. The researcher purposely attempted to approach men of ethnic minority background in urban city areas of South Wales, yet it was difficult to recruit any to take part. Experiences of distress and practices of help-seeking may differ for men from ethnic minority communities, and it would be beneficial for future research to concentrate on the recruitment of, and exploration into, the help-seeking practices of black and ethnic minority men. Despite this limitation, the findings presented extends the field in providing nuanced insights into men’s broader mental health and aspects of men’s help-seeking experiences and pathways in everyday life, including those who may or may not have received a formal clinical diagnosis.

## Data Availability

The original contributions presented in the study are included in the article/Supplementary Material, further inquiries can be directed to the corresponding author.

## References

[B1] Addis M. E. Mahalik J. R. (2003). Men, Masculinity, and the Contexts of Help Seeking. Am. Psychol. 58 (1), 5–14. 10.1037/0003-066X.58.1.5 12674814

[B2] Braun V. Clarke V. (2006). Using Thematic Analysis in Psychology. Qual. Res. Psychol. 3 (2), 77–101. 10.1007/978-1-4614-5583-7_31110.1191/1478088706qp063oa

[B3] Brownhill S. Wilhelm K. Barclay L. Schmied V. (2005). 'Big Build': Hidden Depression in Men. Aust. N. Z. J. Psychiatry 39 (10), 921–931. 10.1080/j.1440-1614.2005.01665.x 16168020

[B4] Chandler A. (2012). “Exploring the Role of Masculinities in Suicidal Behaviour,” in Suicide and Society: Why disadvantaged men in midlife die by suicide. Editors Wyllie C. Platt S. Brownlie J. Chandler A. Connolly S. Evans R. (Surrey, UK. Available at: https://media.samaritans.org/documents/men-suicide-society-samaritans-2012.pdf (Accessed April, , 2019).

[B41] Chandler A. (2021). Masculinities and Suicide: Unsettling ‘Talk’ as a Response to Suicide in Men. Crit. Pub. Health, 1–10. 10.1080/09581596.2021.1908959

[B5] Cheshire A. Peters D. Ridge D. (2016). How Do We Improve Men's Mental Health via Primary Care? an Evaluation of the Atlas Men's Well-Being Pilot Programme for Stressed/distressed Men. BMC Fam. Pract. 17, 13. 10.1186/s12875-016-0410-6 26831720 PMC4736718

[B7] Connell R. W. (1995). Masculinities. Cambridge: Polity Press.

[B8] Connell R. W. (2005). Masculinities. Cambridge: Polity Press.

[B9] Connell R. W. Messerschmidt J. W. (2005). Hegemonic Masculinity. Gend. Soc. 19 (6), 829–859. 10.1177/0891243205278639

[B10] Courtenay W. H. (2000). Constructions of Masculinity and Their Influence on Men's Well-Being: a Theory of Gender and Health. Soc. Sci. Med. 50, 1385–1401. 10.1016/S0277-9536(99)00390-1 10741575

[B11] Courtenay W. (2003). Key Determinants of the Health and Well-Being of Men and Boys. Int. J. Men's Health 2, 1–30. 10.1037/a002982610.3149/jmh.0201.1

[B12] Emslie C. Ridge D. Ziebland S. Hunt K. (2006). Men's Accounts of Depression: Reconstructing or Resisting Hegemonic Masculinity. Soc. Sci. Med. 62, 2246–2257. 10.1016/j.socscimed.2005.10.017 16289741

[B13] Farrimond H. (2012). Beyond the Caveman: Rethinking Masculinity in Relation to Men's Help-Seeking. Health (London) 16 (2), 208–225. 10.1177/1363459311403943 21602248

[B39] Galdas P. Cheater F. Marshall P. (2005). Men and Health Help-Seeking Behaviour: Literature Review. J. Adv. Nurs. 49 (6), 616–623. 10.1111/j.1365-2648.2004.03331 15737222

[B14] Holland J. C. Bultz B. D. (2007). The NCCN Guideline for Distress Management: a Case for Making Distress the Sixth Vital Sign. J. Natl. Compr. Canc Netw. 5 (1), 3–7. 10.6004/jnccn.2007.0003 17323529

[B15] Jeffries M. Grogan S. (2012). 'Oh, I'm Just, You Know, a Little Bit Weak Because I'm Going to the Doctor's': Young Men's Talk of Self-Referral to Primary Healthcare Services. Psychol. Health 27 (8), 898–915. 10.1080/08870446.2011.631542 22149462

[B16] Johnson J. L. Oliffe J. L. Kelly M. T. Galdas P. Ogrodniczuk J. S. (2012). Men's Discourses of Help-Seeking in the Context of Depression. Sociol. Health Illn 34 (3), 345–361. 10.1111/j.1467-9566.2011.01372.x 21707661

[B17] Keohane A. Richardson N. (2018). Negotiating Gender Norms to Support Men in Psychological Distress. Am. J. Mens Health 12, 160–171. 10.1177/1557988317733093 29019282 PMC5734549

[B18] Kiselica M. S. Englar-Carlson M. (2010). Identifying, Affirming, and Building upon Male Strengths: the Positive Psychology/positive Masculinity Model of Psychotherapy with Boys and Men. Psychotherapy (Chic) 47 (3), 276–287. 10.1037/a0021159 22402085

[B19] Leininger M. M. (1985). “Ethnography and Ethno-Nursing: Models and Modes of Qualitative Data Analysis,” in Qualitative Research Methods in Nursing. Editor Leininger M. M. (Orlando, FL: Grune & Stratton), 33–72.

[B20] Möller-Leimkühler A. M. (2002). Barriers to Help-Seeking by Men: a Review of Sociocultural and Clinical Literature with Particular Reference to Depression. J. Affect Disord. 71, 1–9. 10.1016/S0165-0327(01)00379-2 12167495

[B21] O’Brien R. Hunt K. Hart G. (2005). 'It's Caveman Stuff, but that Is to a Certain Extent How Guys Still Operate': Men's Accounts of Masculinity and Help Seeking. Soc. Sci. Med. 61, 503–516. 10.1016/j.socscimed.2004.12.008 15899311

[B22] Office for National Statistics (ONS) (2020). Suicides in the UK: 2019 Registrations, Statistical Bulletin. [Online] Available at: https://www.ons.gov.uk/peoplepopulationandcommunity/birthsdeathsandmarriages/deaths/bulletins/suicidesintheunitedkingdom/2019registrations (Accessed May, 2021).

[B23] Oliffe J. L. Kelly M. T. Johnson J. L. Bottorff J. L. Gray R. E. Ogrodniczuk J. S. (2010). Masculinities and College Men's Depression: Recursive Relationships. Health Sociol. Rev. 19 (4), 465–477. 10.1016/j.socscimed.2004.12.00810.5172/hesr.2010.19.4.465

[B25] Rickwood D. Deane F. P. Wilson C. J. Ciarrochi J. (2005). Young People's Help-Seeking for Mental Health Problems. Aust. E-Journal Adv. Ment. Health 4, 218–251. 10.5172/jamh.4.3.218

[B28] Robertson S. (2007). Understanding Men and Health: Masculinities, Identity and Wellbeing. Buckingham: Open University Press.

[B30] Rogers A. Pilgrim D. (2010). A Sociology of Mental Health and Illness (4e). Maidenhead: Open University Press.

[B31] McManus S. Bebbington P. Jenkins R. Beugha T. (Editors) (2016). Mental health and wellbeing in 721 England: Adult Psychiatric Morbidity Survey 2014. Leeds: NHS Digital. Available at: https://assets.publishing.service.gov.uk/government/uploads/system/uploads/attachment_data/file/556596/apms-2014-full-rpt.pdf (Accessed April, 2019).

[B32] Seidler Z. E. Dawes A. J. Rice S. M. Oliffe J. L. Dhillon H. M. (2016). The Role of Masculinity in Men's Help-Seeking for Depression: A Systematic Review. Clin. Psychol. Rev. 49, 106–118. 10.1016/j.cpr.2016.09.002 27664823

[B33] Sierra Hernandez C. A. Han C. Oliffe J. L. Ogrodniczuk J. S. (2014). Understanding Help-Seeking Among Depressed Men. Psychol. Men Masculinity 15 (3), 346–354. 10.1037/a0034052

[B34] Sieverding M. Matterne U. Ciccarello L. (2010). What Role Do Social Norms Play in the Context of Men's Cancer Screening Intention and Behavior? Application of an Extended Theory of Planned Behavior. Health Psychol. 29, 72–81. 10.1037/a0016941 20063938

[B40] Smith L. K. Pope C. Botha J. L. (2005). Patients’ Help-Seeking Experiences and Delay in Cancer Presentation: A Qualitative Synthesis. Lancet 366 (9488), 825–831. 10.1016/S0140-6736(05)67030-4 16139657

[B36] Strauss A. Corbin J. (1990). “Open Coding,” in Basics of Qualitative Research: Grounded Theory Procedures and Techniques. Editors Strauss A. Corbin J. (Thousand Oaks, CA: Sage), 101–121.

[B37] Valkonen J. Hänninen V. (2012). Narratives of Masculinity and Depression. Men and Masculinities 16 (2), 160–180. 10.1177/1097184X12464377

[B38] Wenger L. M. (2011). Beyond Ballistics: Expanding Our Conceptualization of Men's Health-Related Help Seeking. Am. J. Mens Health 5 (6), 488–499. 10.1177/1557988311409022 21659353

